# Antigenic and immunogenic evaluation of permutations of soluble hepatitis C virus envelope protein E2 and E1 antigens

**DOI:** 10.1371/journal.pone.0255336

**Published:** 2021-07-30

**Authors:** Jannick Prentoe, Christoph M. Janitzek, Rodrigo Velázquez-Moctezuma, Louise Goksøyr, Rebecca W. Olsen, Margherita Fanalista, Elias H. Augestad, Susan Thrane, Anne F. Pihl, Judith M. Gottwein, Adam F. Sander, Jens Bukh

**Affiliations:** 1 Faculty of Health and Medical Sciences, Department of Immunology and Microbiology, Copenhagen Hepatitis C Program (CO-HEP), University of Copenhagen, Copenhagen, Denmark; 2 Department of Infectious Diseases, Hvidovre Hospital, Hvidovre, Denmark; 3 Faculty of Health and Medical Sciences, Department of Immunology and Microbiology, Centre for Medical Parasitology (CMP), University of Copenhagen, Copenhagen, Denmark; 4 Department of Infectious Diseases, Rigshospitalet, Copenhagen University Hospital, Copenhagen, Denmark; Instituto Butantan, BRAZIL

## Abstract

Yearly, about 1.5 million people become chronically infected with hepatitis C virus (HCV) and for the 71 million with chronic HCV infection about 400,000 die from related morbidities, including liver cirrhosis and cancer. Effective treatments exist, but challenges including cost-of-treatment and wide-spread undiagnosed infection, necessitates the development of vaccines. Vaccines should induce neutralizing antibodies (NAbs) against the HCV envelope (E) transmembrane glycoprotein 2, E2, which partly depends on its interaction partner, E1, for folding. Here, we generated three soluble HCV envelope protein antigens with the transmembrane regions deleted (i.e., fused peptide backbones), termed sE1E2 (E1 followed by E2), sE2E1 (E2 followed by E1), and sE21E (E2 followed by inverted E1). The E1 inversion for sE21E positions C-terminal residues of E1 near C-terminal residues of E2, which is in analogy to how they likely interact in native E1/E2 complexes. Probing conformational E2 epitope binding using HCV patient-derived human monoclonal antibodies, we show that sE21E was superior to sE2E1, which was consistently superior to sE1E2. This correlated with improved induction of NAbs by sE21E compared with sE2E1 and especially compared with sE1E2 in female BALB/c mouse immunizations. The deletion of the 27 N-terminal amino acids of E2, termed hypervariable region 1 (HVR1), conferred slight increases in antigenicity for sE2E1 and sE21E, but severely impaired induction of antibodies able to neutralize *in vitro* viruses retaining HVR1. Finally, comparing sE21E with sE2 in mouse immunizations, we show similar induction of heterologous NAbs. In summary, we find that C-terminal E2 fusion of E1 or 1E is superior to N-terminal fusion, both in terms of antigenicity and the induction of heterologous NAbs. This has relevance when designing HCV E1E2 vaccine antigens.

## Introduction

Global HCV prevalence varies geographically from about 0.5% to 2.3%, with nearly 2 million new acute infections annually and a chronicity rate of 55–85% [1]. Chronic HCV infection dramatically increases life-time risk of liver-related morbidities, including liver cirrhosis and hepatocellular carcinoma [2,3]. These long-term effects result in an estimated 400,000 deaths globally every year. Effective direct-acting antivirals are available, but due to several factors, including frequent undiagnosed infection (up to 80%) and cost-of-treatment, there is an urgent need for a prophylactic vaccine [4].

**Fig 1:**
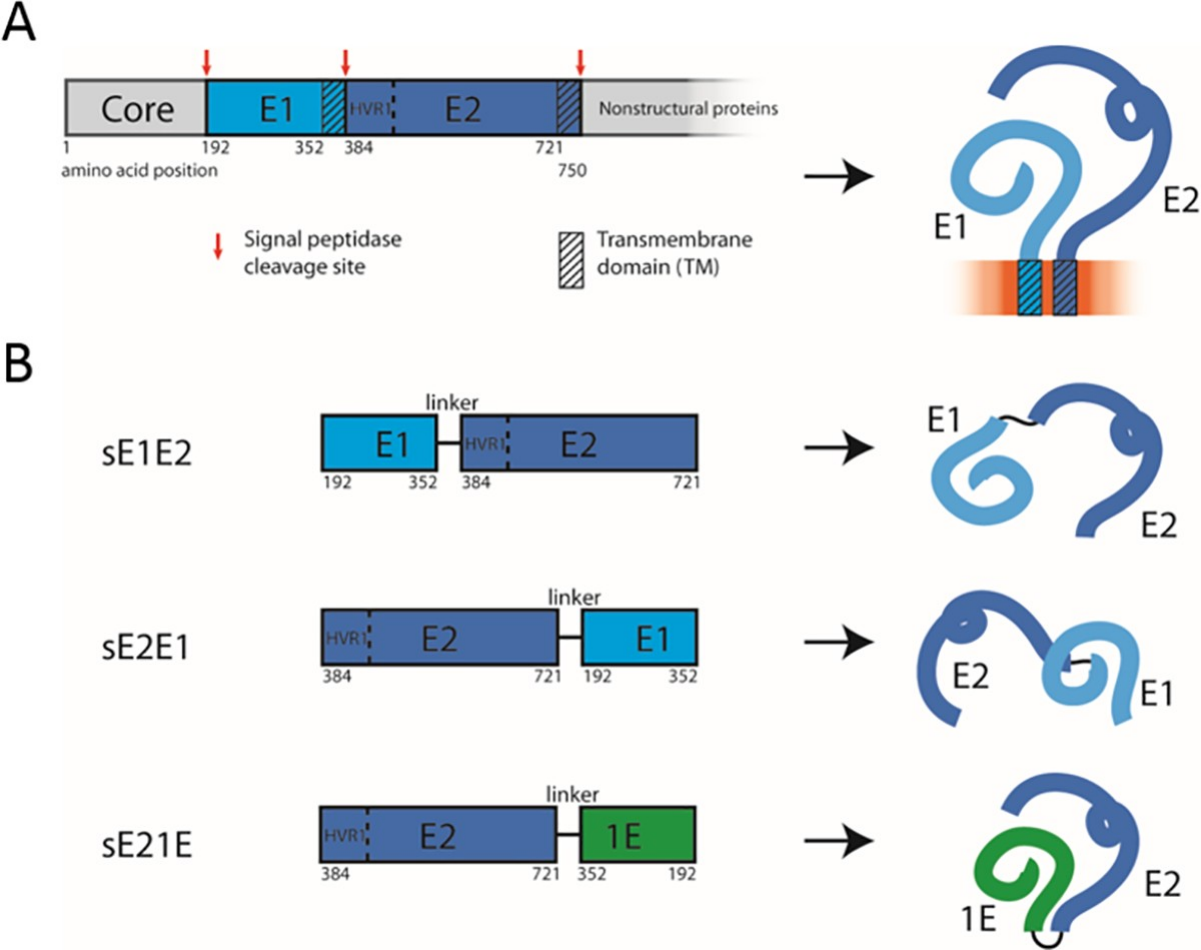
Schematic representations of HCV envelope proteins E1 and E2 as well as soluble permutated variants. A) Left panel, bar representation of the HCV polyprotein, focused on the structural proteins. E1 is followed by E2 and both are cleaved at the end of their respective transmembrane domains (TM) by signal peptidase. Numbering refers to the amino acid sequence of the J6 polyprotein (Genbank # AF177036). HVR1, hypervariable region 1. Right panel, line representation of folded E1/E2 heterodimers embedded in a membrane. B) Left panel, bar representation of permutated, TM-deleted HCV envelope protein variants sE1E2, sE2E1, and sE21E (shown with a linker sequence, GGSAWSHPQFEKGG, between the E1 and E2 constituents). To illustrate how envelope protein variants were derived, the amino acid numbering refers to their numbered positions as shown in panel A.

Six epidemiologically relevant HCV genotypes exist and their RNA genomes differ by 30–33% both at the nucleotide and amino acid level [3]. This sequence diversity is particularly pronounced in the HCV envelope transmembrane proteins E1 and E2, which form the E1/E2 heterodimer that interacts with several cellular co-receptors during entry and is the target of neutralizing antibodies (NAbs) [5,6]. The highest sequence variation of the entire genome is typically found at the 27 N-terminal amino acids of E2, termed hypervariable region 1 (HVR1; Fig 1A), which serves important roles in both NAb evasion and receptor interactions during entry [6–8]. Furthermore, E2 is a primary target of conformational NAbs during natural infection with HCV [9]. It has been suggested by us and others that deleting HVR1 could result in superior vaccine antigens [10], but the deletion of HVR1 alone has, so far, not yielded great increases in induction of cross-reactive NAbs [11–13].

Studies have indicated that E1 folding is dependent on E2 [14,15], whereas the dependence of E1 for proper folding of E2 seemed less certain [15,16]. More recently, it was suggested that E2 folding is, in fact, aided by E1 [17], which may also be supported by reports of antibodies that do not recognize E1 or E2, but only the heterodimer E1/E2 [18–20]. So far, it has proven difficult to extract native E1/E2 complexes from cells and, in fact, the nature of such complexes remains unknown. This has, in part, prompted the many varied attempts to produce forms of transmembrane (TM)-truncated, soluble E1 (sE1) and E2 (sE2) fused via the peptide backbone. Specifically, sE1 and sE2 have been fused both as sE1E2 [21] (C-terminus of sE1 fused directly with N-terminus of sE2) and in a permutated form, sE2E1 [22]. These fused HCV envelope proteins present stable secondary structures and are recognized by antibodies from HCV patients [22]. The antigenicity of sE1E2 was shown to be improved by introducing a linker (GGSAWSHPQFEKGG; abbreviated “L” in sE1E2_L_) between sE1 and sE2, but the antigen still elicited low amounts of HCV NAbs when used for immunization of mice [23].

Here, we aimed to devise a structurally improved HCV E1E2 protein antigen by generating and comparing three variants of backbone fused, soluble (i.e. transmembrane deleted) envelope protein: N-terminal fusion of sE1 to sE2 termed sE1E2 and C-terminal fusion of sE1 or s1E to sE2 termed sE2E1 and sE21E, respectively (Fig 1B). sE21E encodes an inverted sE1 sequence (s1E; inversion at the amino acid level), which orients the membrane-proximal residues of the E1 ectodomain close to the analogous membrane-proximal residues of E2. We hypothesized that this might result in a more native interaction between TM-truncated E1 and E2, and thus could improve the presentation of native E2 epitopes. The ability of s1E to obtain a native E1 fold, when backbone-fused with its folding partner E2, is uncertain. However, previous studies showed that inverted peptides may fold like the original non-inverted peptides, or more likely their mirror image structures [24–26]. Here, we found that C-terminal fusion to sE2 for sE2E1 and especially for sE21E conferred superior E2-specific antigenicity compared with N-terminal sE2 fusion for sE1E2 antigens. Interestingly, performing *in vitro* neutralization studies using cell culture infectious HCV (HCVcc), we found that sE21E also induced higher levels of heterologous (cross-genotype reactive) NAbs compared to sE2E1 and especially to sE1E2_L_ in immunized mice. While sE21E did not induce higher levels of heterologous NAbs than sE2, it may be relevant in future HCV subunit vaccine designs to consider C-terminal fusions to sE2 as opposed to N-terminal fusion.

## Materials and methods

### Expression plasmid generation

HCV sequences used for making envelope protein expression plasmids were of the HCV isolate J6 (genotype 2a; [27]), specifically the ectodomains of envelope protein E1 (amino acid position 192–352) and E2 (amino acid position 384–721). Constructs were generated using standard molecular cloning techniques with the exception of inverted sE1 sequence, the s1E sequence, which was synthesized *de novo* (Genscript), including the reintroduction of four N-linked glycosylation sites by the substitution V43T and Serine insertion at position 4, 18 and 115 in the original sE1 sequence (corresponding to substitution V121T and Serine insertions at position 50, 146, 159 in the s1E sequence). These substitutions restored the canonical N-linked glycosylation site motif (N-X-S/T). The sequences were ligation cloned into a modified pCEP4 vector (ThermoFischer). All constructs that had sE1 at the N-terminus (sE1E2 backbone) had methionine followed by the 35 C-terminal residues of Core as a leader sequence, which includes a signal peptidase cleavage site to ensure proper protein processing. Similarly, all constructs that had sE2 at the N-terminus (sE2E1, sE21E and sE2) had methionine followed by the 35 C-terminal residues of E1 as a leader sequence, which includes a signal peptidase cleavage site to ensure proper protein processing.

### Immunostaining of transfected cells using AR3A

HEK 293T cells were plated at 8 x 10^5 cells per well in 6-well plates 24 h prior to transfection. Cells were transfected with plasmids encoding HCV envelope protein variants using lipofectamine2000 (Invitrogen) as described [28]. Following a 4 hour transfection the cells were immediately replated at 20,000 cells per well in 8-well slides, and incubated for an additional 48 h. Cells were fixed with paraformaldehyde at room temperature for 15 min, permeabilized using 0.5% digitonin, and stained using primary human monoclonal antibody targeting the E2 conformational epitope, AR3A, as described [29]. AR3A binding was visualized using Alexa 488 goat anti-human (Invitrogen) as secondary antibody with a Hoechst 33342 (Molecular Probes) nucleic counterstain.

### HEK 293-T cells transfection

HEK 293-T cells were plated at 6 x 10^5^ cells per well in 6-well plates and incubated overnight at 37°C and 5% CO_2_. Cells were transfected with plasmids encoding the expression of sE1E2_L_, sE2E1 and sE21E with and without HVR1 using lipofectamine2000 (Invitrogen), as per the manufacturer’s instructions. After 6 hours, medium was replaced with DMEM supplemented with 10% fetal calf serum and penicillin/streptomycin, before overnight incubation at 37°C, 5% CO_2_. 72 hours post transfection, cells supernatants were harvested, filtered and stored at -80°C.

### Native-PAGE western blotting of transfected cell supernatant

HEK 293T cell supernatants were diluted in NativePAGE sample buffer with 5% G-250 sample additive (Invitrogen) and loaded in a NativePAGE 4–16% Bis-Tris gel (Invitrogen). Gel electrophoresis was carried out at 150V in Dark Blue Buffer (5% NativePAGE 20X Cathode Additive supplemented native running buffer, Invitrogen) until the dye front migration reached 1/3 of the gel. Gel electrophoresis was continued in Light Blue Buffer (0.5% NativePAGE 20X Cathode Additive supplemented native running buffer, Invitrogen) until protein migration reached the end of the gel. After gel electrophoresis, proteins were transferred to a PVDF membrane by western blotting at 25V for 1 hour. After transfer, the proteins were fixed by incubating the membrane with 8% acetic acid for 15 minutes. The membrane was rinsed with deionized water, air-dried and incubated with ethanol until dye was removed. The membrane was incubated in blocking buffer (PBS 1% bovine serum albumin 0.2% skim milk powder) for 30 minutes. The proteins were probed using E2-specific AR3A antibody [30] and HRP-conjugated F(ab’)2-goat anti-human IgG Fc secondary antibody (Invitrogen) and visualized by chemiluminescence.

### SDS-PAGE western blotting of transfected cell supernatant

Cells supernatants were diluted in NuPAGE LDS sample buffer and NuPAGE reducing agent (Invitrogen), heated at 70°C for 10 minutes, and loaded in a NuPAGE 4–12% Bis-Tris Gels (Invitrogen). Gel electrophoresis was carried out at 200V for 50 minutes in MOPS SDS running buffer containing NuPAGE antioxidant (Invitrogen). After gel electrophoresis proteins were transferred to a PVDF membrane by western blotting at 30V for 1 hour. After transfer, the membrane was rinsed with deionized water and incubated in blocking buffer (PBS 1% bovine serum albumin and 0.2% milk) for 30 minutes. The proteins were probed using E2-specific AP33 antibody [31] and HRP-conjugated goat anti-mouse IgG (H+L) secondary antibody (Invitrogen) and visualized by chemiluminescence.

### Protein expression and purification

Protein was expressed in HEK293-F or HEK293-EBNA1 cells. For purification purposes, all constructs included either a Tobacco etch virus cleavage sequence (TEV)-Twin-Streptavidin tag (LENLYFQSASWSHPQFEKGGGSGGGSGGGSWSHPQFEK) or a TEV-hexa-Histidine tag (LENLYFQSASHHHHHH) at the C-terminus. HEK293-F cells or HEK293-EBNA1 were cultured in suspension by rotation (150rpm, humidified, 37°C, 5% CO_2_) using FreeStyle^TM^ 293 Expression Medium (Gibco). The transfection reagent was prepared by diluting 100μg endotoxin free plasmid in OptiPro^TM^ SFM (Gibco) and subsequently mixing the plasmid with FreeStyle ^TM^ Max Reagent (Gibco) that had been diluted in OptiPro^TM^ SFM (Gibco). Following a 10 minutes incubation step the transfection reagent was added dropwise to HEK293-F cells (1.0x10^6^ cells/ml). The cells were further incubated in suspension (as above) and proteins were expressed and secreted to the media over a course of 5–6 days. The supernatant was harvested by centrifuging the cells 4000xg for 10 minutes. The media was exchanged to PBS buffer (8mM Na_3_PO_4_, 1.5mM K_3_PO_4_, 137mM NaCl, 2.7mM KCl, pH 7.2) by dialyzing (6-8kDa Molecular Weight Cut Off, (MWCO) Spectrum Labs). Purification was either done using Strep-Tactin column purification or by using Ion Metal Affinity Chromatography (IMAC). Following purification, imidazole or desthiobiotin was removed by dialysis (Spectrum Labs) against PBS buffer.

### Enzyme linked immunosorbent assays (ELISAs)

96 well maxisorp plates (NUNC) were coated overnight using 80 μl/well of a 10 μg/ml PBS suspension of protein of interest and negative wells incubated with PBS only. All wells were blocked by a 1-hour incubation at room temperature using a 1% suspension of bovine serum albumin (Sigma) in PBS with 0.1% Tween20 (Sigma; PBS/Tween). Mouse antibodies purified from immunized animals or human monoclonal antibodies AR2A, AR3A, CBH4G, CBH5, CBH7, HC84.26 or IGH520 against the HCV envelope proteins [30,32–34] were used as primary antibodies. These antibodies were either incubated in the wells at a fixed dose of 10 μg/ml or in a 4-fold dilution series starting at 10 μg/ml. Negative control antibody was b6 [30]. Wells were washed three times in PBS/Tween and incubated for 1 hour either with horse radish peroxidase (HRP)-coupled secondary anti-human antibody or HRP-coupled secondary anti-mouse antibody, as appropriate. Following three additional PBS/Tween washes, antibody binding was assessed by adding TMB substrate (Sigma), stopping the reaction following 3–10 minutes and measuring the colorimetric signal at 450 nm.

### Mouse immunizations

Mouse immunization studies were conducted in order to evaluate the efficacy of proteins expressed from the different HCV constructs as vaccine antigens. In all studies, 7–10 week old female BALB/c mice were immunized intramuscularly in the thigh at day 0, 21 and 42. Blood samples were taken at day 14, 35 and 56. In the first animal trial, used to screen for the optimal antigen, a total of 18 mice were randomly allocated into six groups (n = 3), which received a dose (4–10μg) of either E1E2_L_, E2E1, E21E or their corresponding constructs without HVR1 (formulated in Freunds Incomplete Adjuvant, Invivogen), per immunization. In a subsequent study, a total of 16 BALB/C mice were randomly allocated into two groups (n = 8), which received 5μg of either sE21E or sE2 (formulated in AddaVax, Invivogen) per immunization. Besides gender and age, no other inclusion/exclusion criteria were set for the mice. No protocol for these experiments were registered elsewhere prior to the study. All study data is included in the manuscript and no confounders were controlled for.

### Mouse antibody purification and quantification

In order to conduct the neutralization experiments, mouse antibodies were purified from cardiac bleed sera (day 56). Mouse antibodies were purified using either Amicon pro-affinity purification columns Protein G (Merck) or Polyprep chromatography columns (Bio-Rad) loaded with Gammabind Plus Sepharose (GE Healthcare), as per the manufacturer’s instructions. Briefly, the columns were washed with PBS and the mouse serum samples were filtered (0.2μm) and passed through columns multiple times for binding. The columns were washed as per the manufacturer’s instructions and mouse antibodies were eluted in weak acid (pH 2.5–2.7). pH was neutralized to a neutral pH using 1M Tris-HCl buffer. Eluted antibodies from the Amicon columns were buffer exchanged on the columns into PBS, concentrated using Vivaspin 6 columns (MWCO 50kDa) and the antibody concentrations were measured using Mouse IgG (Immunoglobulin G) Total Ready-Set-Go (Affymetrix) ELISA as per the manufacturer’s instructions. Eluted antibodies from the Polyprep columns were dialyzed (MWCO 12-14kDa, Spectrum Labs) against PBS and concentrated using Vivaspin 6 columns, MWCO 50kDa, and the antibody concentrations were quantified using the IgG setting on a Nanodrop2000 (Thermo Scientific).

### *In vitro* neutralization assay

7x10^3^ Huh7.5 cells [35] were plated per well in poly-D-lysine 96-well plates and incubated for 24 hours. The following day, a dilution series of mouse-derived antibodies was made using DMEM (Gibco) supplemented with 10% fetal calf serum (Sigma) and penicillin/streptomycin (Sigma) (full medium). This was incubated in a total volume of 10 μl with JFH1-based Core-NS2 recombinant HCVcc stocks (H77 (genotype 1a), H77_ΔHVR1_, J4 (genotype 1b), J6 (genotype 2a), J6_ΔHVR1_ or S52 (genotype 3a) [7,36–39]), corresponding to a final read-out of 50–200 FFU per well. The virus/antibody mixes were, along with eight replicates of virus only, incubated for 1.5 hours at 37°C before addition of an additional 20 μl of full medium and incubation with Huh7.5 cells for 2.5 hours at 37°C in 5% CO_2_. Cells were washed, and fresh medium was added prior to incubation for a total infection time of 48 hours before fixation. Following fixation, the cells were incubated with anti-mouse Fab fragments (Jackson Immuno Research) to block the Fc region of the mouse antibodies. Staining and counting of the number of FFUs were done as described [40,41], with primary NS5A-specific antibody 9E10 [39] followed by secondary antibody Anti-mouse IgG, Horseradish Peroxidase (Amersham Biosciences) and visualized by DAB staining (VWR). The data were normalized to 8 replicates of virus only and analyzed using three or four parameters curve fitting in GraphPad Prism 8.0.0, bottom set to 0, top set to 100.

### Ethical statement

All animal experiments were conducted in accordance with national Danish guidelines and were approved by the National Animal Experiments Inspectorate (Dyreforsøgstilsynet, license no. 2018-15-0201-01541). Mice were housed in an AAALAC accredited facility in accordance with good animal practice as defined by FELASA. Mice were monitored for adverse effects throughout the study and finally euthanized by cervical dislocation.

## Results

### Generating permutated soluble E1 and E2 HCV recombinant protein expression plasmids

We generated plasmids for mammalian protein expression encoding three different permutations of transmembrane domain-deleted (e.g. solubilized ectodomains) HCV envelope proteins, sE1 and sE2 (Fig 1A), of the genotype 2a isolate, J6 [27,39]. These constructs were named sE1E2, sE2E1, and sE21E and represent sE1 followed by sE2, sE2 followed by sE1 and sE2 followed by sequence inverted sE1 (i.e. s1E), respectively. The four canonical N-linked glycosylation motifs (N-X-S/T sites) of E1 were reintroduced at the appropriate sites in the 1E sequence by restoring the motif through serine insertion or a valine to threonine substitution (See Methods). The deletion of the transmembrane domains had the additional effect of removing the signal peptidase cleavage sites, resulting in a fused peptide backbone. To minimize potential steric constraints on E1 and E2 orientation imposed by the fused backbone, we also introduced a linker between E1 and E2, consisting of the amino acid sequence GGSAWSHPQFEKGG, which has previously been used successfully as a linker between sE1 and sE2 (Fig 1B). This linker was previously described to increase antigenicity of a similarly backbone-fused E1E2 recombinant protein from the genotype 1a isolate, H77 [23]. Thus, we also generated sE1E2_L_, sE2E1_L_, and sE21E_L_ expression plasmids.

### Binding of HCV-specific NAbs to permutated E1 and E2 HCV envelope proteins

**Fig 2:**
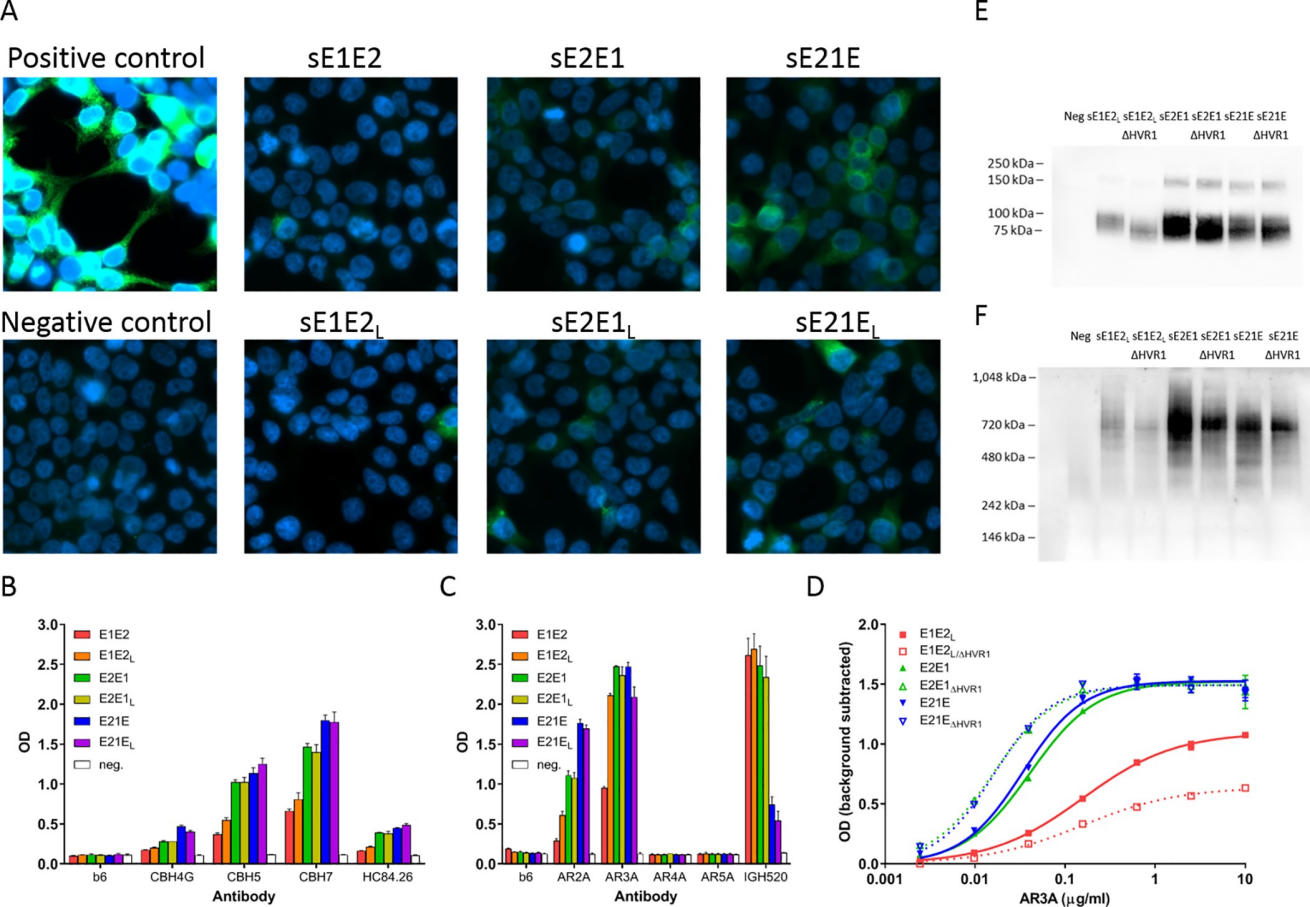
Antigenicity testing by ELISA of recombinant sE1E2, sE2E1 and sE21E with and without linker (L) between E1 and E2 and/or HVR1. A) HEK 293T cells were transfected with expression vectors for the indicated proteins with or without a linker, GGSAWSHPQFEKGG, and stained using the HCV NAb, AR3A. Positive control is full-length J6 E1E2 (high signal due to full cellular retention via non-truncated TM domains) and negative control is empty vector. B-D) The indicated affinity purified proteins were bound to 96 well maxisorp plates by overnight incubation at 4°C followed by blocking using BSA for 1h. Subsequently the proteins were incubated with (B and C) a single high dose (5 μg/ml) of the indicated human primary anti-HCV envelope antibodies and b6 control antibody or D) a dilution series of the antibody AR3A. Binding was measured by HRP-coupled anti-human antibody followed by colorimetric optical density (OD) reading at 450 nm of the developed TMB substrate signal. E and F) HEK 293T cells were transfected with plasmids encoding expression in mammalian cells of sE1E2_L_, sE2E1 and sE21E with and without HVR1. Following 72h, supernatants were collected and subjected to SDS-PAGE under reducing conditions or native-PAGE (See S1 Raw images for uncropped and unmodified blots). E) Following SDS-PAGE the proteins were transferred onto a PVDF membrane and probed using E2-specific antibody, AP33, which was visualized using chemiluminescence with an HRP-coupled anti-mouse antibody. F) Following native-PAGE the proteins were transferred onto a PVDF membrane and probed using E2-specific antibody, AR3A, which was visualized using chemiluminescence with an HRP-coupled anti-human antibody. Neg is negative control and represents transfection with empty vector.

Using these six expression plasmids (coding for sE1E2, sE2E1, sE21E and the corresponding sE1E2_L_, sE2E1_L_, and sE21E_L_, which include the linker), we transfected HEK 293T cells and stained for correctly folded E2 protein using the HCV NAb, AR3A (Fig 2A). Here, we observed relatively weak binding for all constructs with a trend towards higher signal for sE2E1 and especially sE21E. The weak signal could be caused by efficient release of produced protein as the constructs did not encode TMs. Next, we produced the recombinant proteins in HEK 293Freestyle cells and performed single-step affinity purification on cell culture supernatants using a C-terminal protein strep tag. Protein from supernatants was analyzed by Coomassie staining of SDS-PAGE gels before and after purification confirming affinity purification (Data not shown). To test folding of the six recombinant proteins, the purified proteins were immobilized on 96-well plates for ELISA testing of binding to the human monoclonal anti-E2 antibodies CBH4G (antigenic domain A), CBH5 (antigenic domain B), CBH7 (antigenic domain C), HC84.26 (antigenic domain D), AR2A (antigenic region 2A), AR3A (antigenic region 3A), AR4A (antigenic region 4A), AR5A (antigenic region 5A), all of which, with the partial exception of HC84.26, recognize conformational, non-linear epitopes. An anti-E1 antibody, IGH520, was also used, as well as the negative control antibody, b6. Neither AR4A nor AR5A bound any of the constructs indicating that native E1/E2 complexes were not achieved. The linker improved antibody binding (e.g. antigenicity) of sE1E2, as previously observed for H77 sE1E2 [23], but did not consistently improve antigenicity of sE2E1 or sE21E (Fig 2B and 2C). Interestingly, we also observed that the antigenicity of sE21E was consistently superior to sE2E1, which was consistently superior to sE1E2_L_ (Fig 2B and 2C). The exception to this trend was the E1-specific antibody, IGH520, which expectedly had greatly reduced binding to 1E in sE21E as compared to E1 in sE1E2 and sE2E1 (Fig 2C).

As we have shown that removing HVR1 from E1/E2 on infectious HCVcc dramatically increases NAb sensitivity [7], we tested the effect on antibody binding of removing HVR1 from these recombinant proteins. Thus, we deleted the 27 N-terminal amino acids of E2 (constituting HVR1; position 384–410 in Fig 1) from the sE21E, sE2E1, and sE1E2_L_ constructs. Protein from these constructs were expressed and purified using HEK 293Freestyle cells. Then, we performed a dose-response ELISA against sE1E2_L_, sE2E1, and sE21E with and without HVR1 using AR3A. For sE1E2_L_, in which HVR1 is located within the fused protein (between E1 and E2), the deletion of HVR1 decreased antibody binding. For sE2E1 and sE21E, in which HVR1 is located at the N-terminus, we observed very modest increases in AR3A binding (Fig 2D). To evaluate protein complex size of sE21E, sE2E1, and sE1E2_L_ as well as any impact of HVR1 removal we transfected these constructs into HEK 293T cells and collected supernatants after 72h. We then performed an SDS-PAGE western blot using the HCV NAb, AP33, which recognizes a linear E2 epitope as well as a native-PAGE western blot using the potent neutralizing antibody, AR3A (Fig 2E and 2F). The data confirmed expression and release of soluble proteins with reduced, unfolded protein size around 100 KDa (Fig 2E) and an estimated folded complex size around 700 KDa (Fig 2F).

### HCV-specific NAbs in mice immunized with sE1E2_L_, sE2E1 and sE21E with and without HVR1

**Fig 3:**
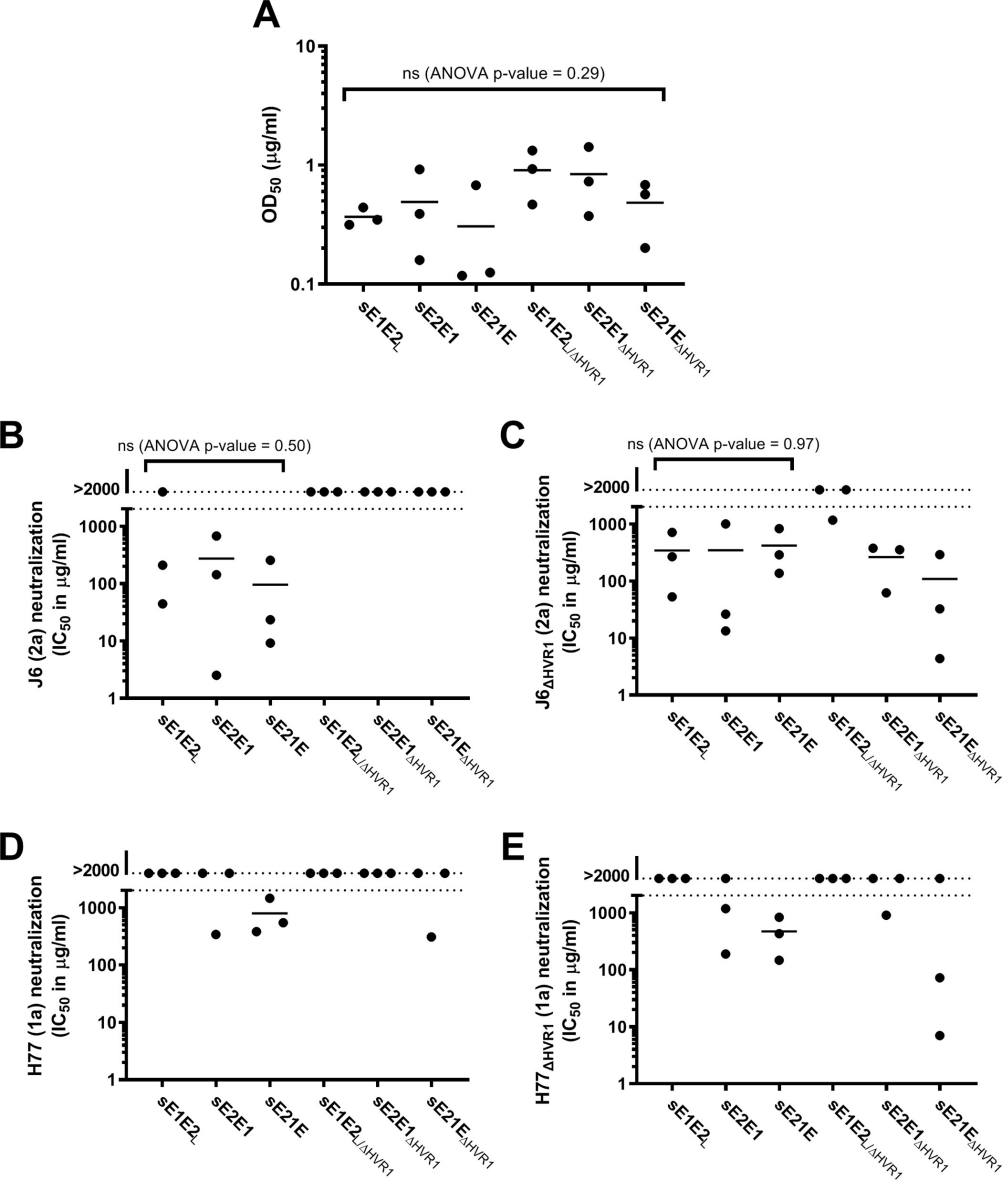
Comparison of immunogenicity and ability to induce HCV NAbs of sE1E2, sE2E1 and sE21E with and without HVR1 in mice. 7–8 week-old BALB/c mice were immunized with the indicated HIS-tagged antigens in Freund’s incomplete adjuvant and subsequently boosted twice with 3 week intervals. 2 weeks after the second boost the animals were euthanized, and a full bleed was taken and allowed to clot to obtain serum. Total mouse antibodies were purified from the serum samples using Protein G affinity chromatography and mouse antibody concentrations in the samples were measured. A) The purified antibody samples were tested for reactivity in ELISAs against the proteins used to immunize the animals. OD_50_ (50% optical density) values were calculated based on four parameter sigmoidal dose-response curves (Graphpad PRISM 8.0.0). HCV NAbs in the purified antibody samples were measured against HCVcc of isolates B) J6 (genotype 2a; autologous neutralization), C) J6_ΔHVR1_, D) H77 (genotype 1a; heterologous neutralization), and E) H77_ΔHVR1_ in dose-response neutralization assays starting at 1000 μg/ml. IC_50_ values were calculated based on four parameter sigmoidal dose-response curves (Graphpad PRISM 8.0.0). If 50% neutralization was not reached at the highest tested concentration, an IC_50_ value was extrapolated if this value was below 2000 μg/ml (assay cut-off). No data points were excluded from the analyses.

We compared the ability of the three proteins sE1E2_L_, sE2E1, and sE21E with and without HVR1 to induce NAbs in mice. Thus, we immunized groups of BALB/c mice (n = 3) with each of the six antigens (purified using a C-terminal hexahistidine (HIS) tag) formulated in Freund’s incomplete adjuvant. Specifically, mice were immunized using a two-week interval prime-boost-boost regimen, and then sacrificed two weeks after the final immunization to obtain a final high-volume bleed. Total mouse Immunoglobulin G (IgG) antibodies were purified from the obtained sera and antibody concentrations were measured. The purified antibodies were initially tested in ELISA for binding to the respective antigens. Although variation was observed, we overall found similar, high levels of antigen-specific antibody responses (Fig 3A), which was confirmed by one-way ANOVA with a p-value of 0.29. Subsequently, we assessed the quality of the responses by using the mouse antibodies to perform dose-response neutralization of a genotype 2a HCVcc (J6; autologous neutralization) and a genotype 1a HCVcc (H77; heterologous neutralization), as well as the HVR1-deleted variants J6_ΔHVR1_ and H77_ΔHVR1_, which have greatly increased NAb sensitivity [7]. Interestingly, despite clear antigenic differences between sE1E2_L_, sE2E1 and sE21E (Fig 2A and 2B), they all elicited autologous NAbs (Fig 3B and 3C). Despite variation between animals, autologous NAb levels were comparable, as was confirmed by one-way ANOVA with a p-value of 0.50 (NAbs against J6; Fig 3B) and 0.97 (NAbs against HVR1-deleted J6; Fig 3C). However, sE1E2_L_ did not elicit measurable levels of heterologous NAbs, even against highly sensitive HVR1-deleted, H77_ΔHVR1_ (Fig 3D and 3E). In addition, sE21E was the only antigen able to elicit measurable autologous and heterologous NAb responses in all three animals in each group (Fig 3B–3E). The HVR1-deleted antigens elicited antibodies that were either non-neutralizing (sE1E2_L/ΔHVR1_) or only neutralized HVR1-deleted viruses (sE2E1_ΔHVR1_ and sE21E_ΔHVR1_), indicating that they primarily targeted epitopes subject to high levels of HVR1-mediated protection. We concluded that sE21E was the most promising antigen for further study.

### Comparison of NAb responses from mice immunized with sE2 or sE21E

**Fig 4:**
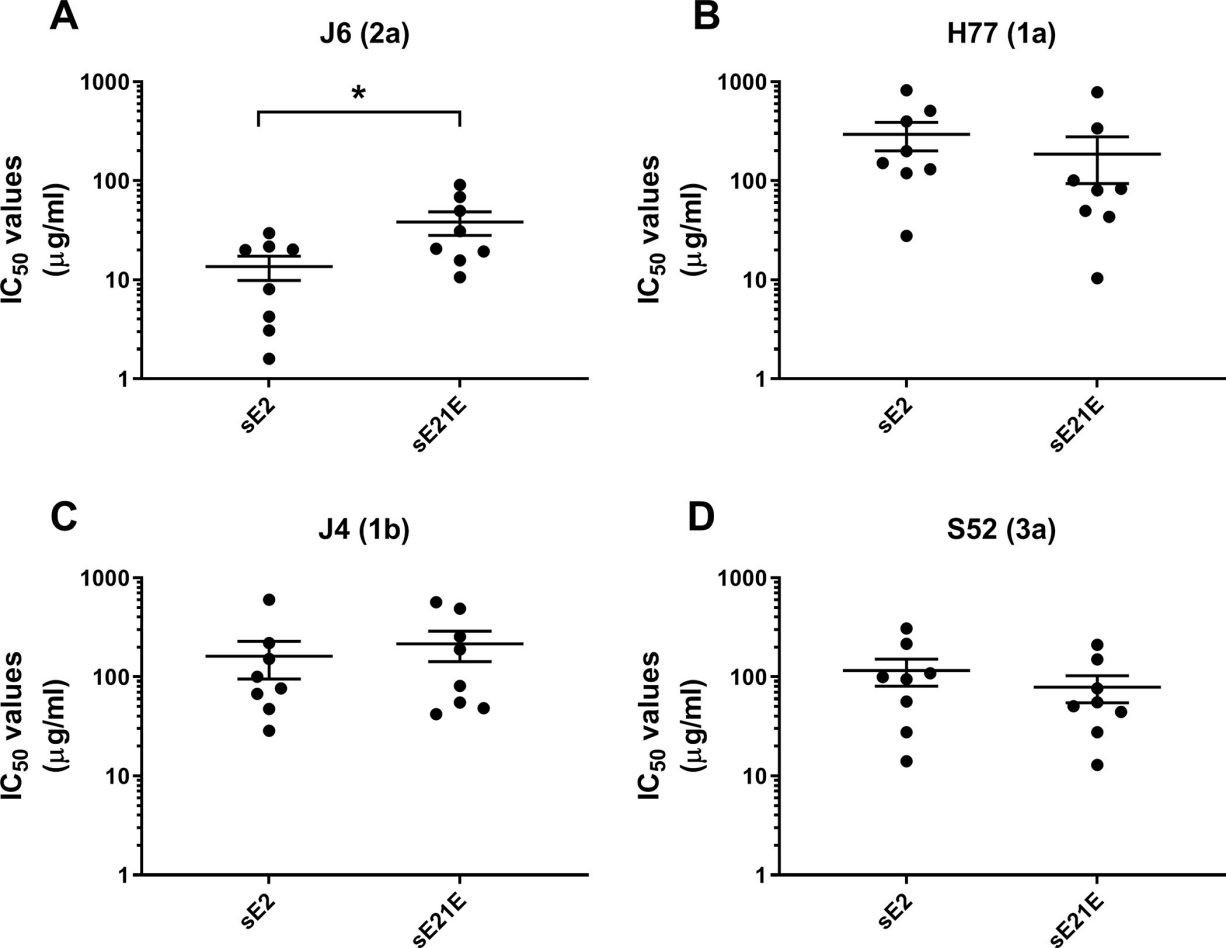
Comparing the ability of sE21E and sE2 to induce HCV NAbs in mice. 8–10 week-old BALB/c mice were immunized with either sE21E or sE2 with HIS tags in AddaVax adjuvant and subsequently boosted twice with 3 week intervals. Mouse antibodies were obtained from full bleeds, as described in Fig 3. HCV NAbs were measured against HCVcc of isolates A) J6 (2a, autologous neutralization), B) H77 (1a, heterologous neutralization), C) J4 (1b, heterologous neutralization), and D) S52 (3a, heterologous neutralization) in dose-response neutralization assays. IC_50_ values were calculated based on four parameter sigmoidal dose-response curves (Graphpad PRISM 8.0.0). Statistical significance of the differences between groups was assessed for each virus in two-tailed, unpaired t-tests at the 95% significance level. *, Neutralization efficacy of mouse antibodies from animals immunized with sE21E or sE2 were statistically significantly different for the indicated virus. No data points were excluded from the analyses.

Most humoral vaccine approaches currently being pursued for HCV employ variants of sE2 to induce NAbs. Thus, we produced J6 sE2 as described above for sE21E. To compare the immunogenicity of sE21E with sE2, we immunized groups of BALB/c mice (n = 8) prime-boost-boost with each antigen. Both vaccines were formulated using AddaVax, which has a similar composition (squalene oil-in-water emulsion) as the clinically approved MF59 adjuvant. Two weeks after the final immunization, animals were sacrificed to obtain a final high-volume bleed. Mouse IgG were purified from the resultant sera and antibody content was quantified. Subsequently, we assessed the quality of the responses by using the mouse antibodies to perform dose-response neutralization against HCVcc of genotype 2a (J6; autologous neutralization) and genotypes 1a (H77), 1b (J4) and 3a (S52) (heterologous neutralization). On average, sE2 elicited a statistically significant ~3-fold (unpaired t-test; p-value = 0.0392) higher level of autologous NAbs compared with sE21E (Fig 4A). However, sE2 and sE21E elicited comparable levels of heterologous NAbs (Fig 4B–4D; no statistical differences).

## Discussion

The urgent need for developing an HCV vaccine that protects against chronicity hinges on uncovering ways to produce optimally folded HCV envelope proteins that adequately expose cross-genotype conserved NAb epitopes. It seems clear that E1 and E2 folding are mutually dependent in the context of native E1/E2 complexes. However, it has proven technically difficult to extract and purify such E1/E2 complexes and solubilization by TM truncation has, so far, not resulted in E1E2 envelope chimeras able to induce high levels of heterologous NAbs [23]. Here, we developed three distinct permutations of soluble E1E2 (sE1E2, sE2E1 and sE21E), and by performing ELISAs with HCV-specific conformation-dependent NAbs, we observe increased binding of HCV NAbs to sE21E, compared with sE2E1 and especially sE1E2. This correlated with the ability of sE21E to induce higher levels of cross-genotype reactive NAbs in mouse immunizations compared with sE2E1 and especially sE1E2_L_. Compared with sE2 we find that sE21E induced comparable levels of heterologous NAbs. Our data suggests that C-terminal fusion to sE2 of accessory proteins (in this study sE1 or s1E) is superior to N-terminal fusion. This has relevance for future efforts to develop E1E2 subunit vaccine antigens.

The most common approach to virus vaccine development has historically been whole inactivated or attenuated virus particles, and efforts are ongoing to develop whole particle vaccines for HCV. Studies have shown that mouse immunizations with inactivated particles lead to the induction of NAbs, but NAb titers in immunized animals have so far been modest [42,43]. In addition, despite recent advances in production and downstream processing of inactivated HCVcc [44–47], challenges persist in large-scale HCVcc production. Thus, the use of subunit vaccine approaches is an attractive alternative. While detergent extraction of E1/E2 complexes and subsequent vaccination of humans in a clinical safety trial resulted in the induction of cross-reactive NAbs in some vaccinees [48], purifying native E1/E2 complexes remains another big obstacle. Instead, many subunit vaccine approaches have focused on various truncated, soluble forms of E2 alone [12,49,50]. While both detergent extracted E1/E2 and sE2 are able to induce NAbs in animal immunizations [51], the absence of knowledge regarding the native E1/E2 complex composition renders it unclear whether better NAb responses could be achieved by subunit vaccine approaches in which the antigen more closely resembles such complexes.

One known aspect of E1-E2 interactions is their heterodimeric association in the membrane, mediated through their respective TM regions [52–54]. Consequently, it is expected that the stalk regions of E1 and E2, immediately upstream of the TM regions, also be proximal as they emerge from the membrane and could be involved in critical E1-E2 interactions. Based on this assumption, we attempted to engineer improved folding of sE2 by fusing the C-terminus of sE2 to an inverted E1 amino acid sequence (termed 1E). The effects on protein structure of inverting the E1 sequence at the amino acid level are likely profound for a number of reasons including: ^i^the peptide backbone is not symmetrical, which is particularly prominent for proline sidechain that are covalently attached to the backbone at two positions (J6 sE1 contains 9 proline residues); ^ii^Given that most amino acid sidechains are in the trans configuration, it is likely that even a complete native folding of s1E (compared with sE1) would result in the mirror image structure of sE1; and ^iii^N-linked glycosylation motifs, N-X-S/T, are directional in the primary sequence (J6 sE1 contains 4 N-linked glycosylation motifs). Most of these effects were not amenable to further manipulation, but we did reestablish N-linked glycosylation motifs by minimal site-directed mutagenesis of the 1E sequence.

To our knowledge, there are no studies that involve the inversion of the protein backbone of an entire protein domain or, as done here, the entire ectodomain of a transmembrane protein. However, a number of experimental and modelling studies have suggested that inversion of short and long peptide amino acid backbones can result in folding, which resembles the fold of the original non-inverted peptides [24,26,55]. This has been suggested to apply even for inverted peptide sequences substituted for the original peptide within the context of a complete protein and it was hypothesized to be partly due to a “conserved structural environment” at the relative position of the inverted peptide within the protein [25]. As mentioned, the mutual folding dependency of E1 with E2 has been shown to partially depend on interactions within their respective transmembrane domains [53,54,56] and consequently it seemed likely that the C-terminal parts of the E1 and E2 ectodomains would be proximal as they emerge from the membrane. Thus, we speculated whether inverting the E1 ectodomain sequence (i.e. 1E) in sE21E to position the C-terminal residues of the E1 ectodomain close to the C-terminal residues of the E2 ectodomain might result in a “structural environment” in which folding of sE2 was improved by the proximity of s1E residues that are of relevance in native E1/E2 complex formation. The rationale, as outlined above, makes it intriguing that HCV NAbs generally have increased binding to sE21E, compared with sE2E1 and especially sE1E2, and that sE21E is better at inducing cross-genotype reactive HCV NAbs in mouse immunizations. However, it should be noted that sE21E did not elicit higher levels of heterologous NAbs than sE2 and it is possible that sE1 fused directly to the backbone of sE2 negatively impacts folding of sE2 and that incorrectly folded s1E does not. This negative effect of backbone fusing E1 to E2 is supported by a recent study in which the insertion of a cleavable linker sequences between TM-truncated, soluble E1E2 chimeric proteins assists in native protein interaction [57]. It is interesting that sE2 appears to tolerate C-terminal protein fusion better than N-terminal fusion, which is a useful starting point for engineering sE2 protein variants fused to immunostimulatory proteins, such as CR2 receptor-binding domain (p28), as has been used with some success in Dengue virus vaccine research [58].

Research by us and others showed that the removal of the 27 N-terminal amino acids from E2 (i.e., HVR1) led to increased exposure of NAb epitopes on HCVcc both *in vitro* [7,59] and *in vivo* [8]. Interestingly, this was found to be true for a wide range of non-overlapping epitopes, some of which were cross-genotype conserved and some that were not [10]. However, we observed only modestly increased AR3A NAb binding to HVR1-deleted sE1E2 and sE21E compared to the original antigens retaining HVR1. This was in contrast to the dramatic increases in AR3A neutralization sensitivity observed for HVR1-deleted HCVcc [10]. The explanation for this discrepancy is likely linked to the mechanism of HVR1-mediated NAb protection. We recently showed that HVR1 is not directly occluding underlying epitopes, but rather serves as a critical feature of the E1/E2 complex involved in stabilizing “closed”, difficult-to-neutralize E1/E2 states [60] and in fact the behavior of these E1/E2 states on infectious HCV particles is only poorly recapitulated by sE2 and even by E1/E2 expressed on cells [60,61]. Performing mouse immunizations of these antigens with or without HVR1, we found that HVR1 removal did not lead to an increase in the induction of broadly reactive NAbs. In fact, it resulted in a marked reduction of such antibodies targeting HCVcc retaining HVR1, whereas neutralization against HVR1-deleted HCVcc remained high. This suggests that the removal of HVR1 from vaccine antigens caused the immune system to primarily target otherwise protected epitopes, which for that reason were not effective vaccine targets. These epitopes appeared to be relatively well-conserved, as evidenced by significant neutralization of HVR1-deleted HCVcc of a genotype different from that of the antigen. Others have removed HVR1 from full-length E1E2, as well as from sE2, and found no benefit in terms of the induction of NAbs [11,13]. However, HCV vaccine antigens with truncation of all three E2 hypervariable regions (HVR1, HVR2 and IgVR), resulted in the induction of effective NAbs [12], and could suggest that such additional truncations may improve sE21E vaccine potential.

In conclusion, we show that sE21E and sE2E1 are superior to sE1E2, irrespective of the inclusion of an intra-protein linker sequence. This was true both in terms of HCV NAb binding and the ability to induce cross-reactive HCV NAbs, although it should be pointed out that NAb responses will differ between rodents and humans. However, sE21E did not induce higher levels of heterologous NAbs compared with sE2. Thus, our data suggests that sE2 tolerates C-terminal fusion partners (here sE1 and s1E) better than N-terminal fusion partners, which has implications for future E1E2 subunit vaccine approaches.

## Supporting information

S1 Raw images Uncropped and unmodified western blots related to Fig 2E and 2F. Panels A and B refer to the same SDS PAGE western blot. Panels C and D refer to the same blue native PAGE western blot. A) Blot was exposed for a short duration to visualize marker bands while avoiding over-exposure. B) Blot was exposed for a longer time to better visualize the annotated HCV envelope protein bands. C) As no blue native PAGE western blot marker was available the protein bands from a colorimetric native PAGE marker were marked with a pencil on the blot following transfer, but before ethanol washing. The pencil marks are clear in the colorimetric picture of the blot (panel C) and can be made out as faint white markings in the chemiluminescence capture (panel D). D) folded HCV E2 protein was probed on the blot using the conformational antibody, AR3A.
(PDF)
