# Supplementary figures and images for: Antigenic and immunogenic evaluation of permutations of soluble hepatitis C virus envelope protein E2 and E1 antigens
Source: PLoS One. 2021 Jul 30;16(7):e0255336. doi: 10.1371/journal.pone.0255336 (PMC8323887; doi:10.1371/journal.pone.0255336)

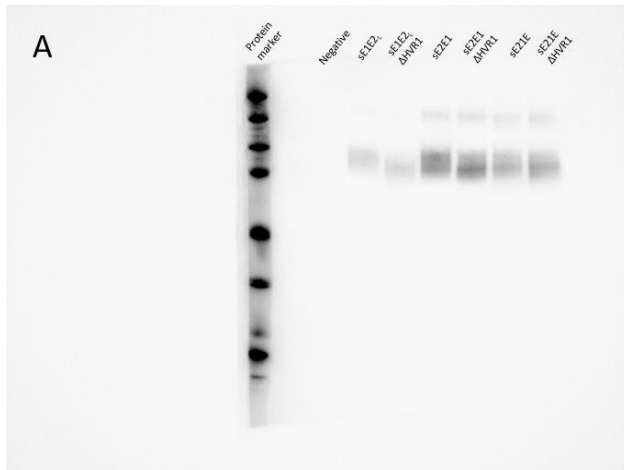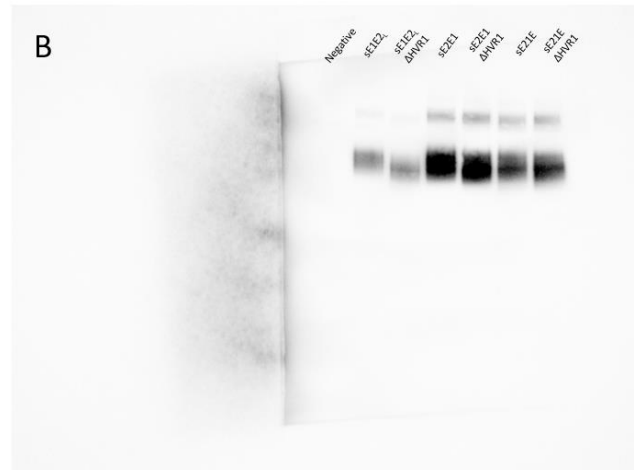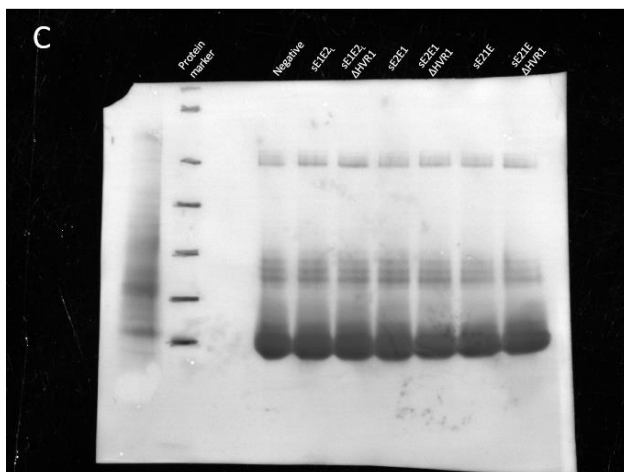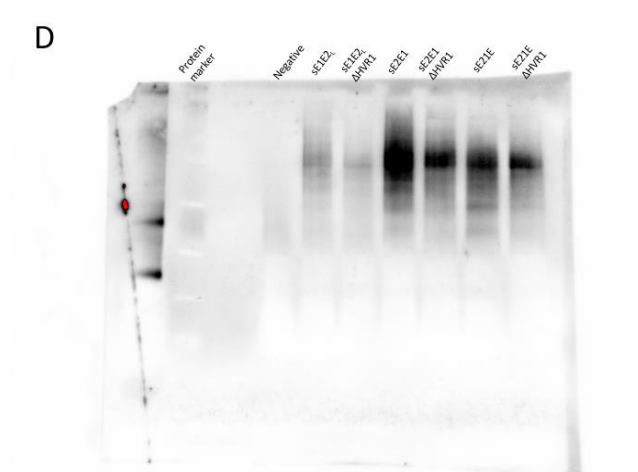

Supplement: S1 Raw images — Panels A and B refer to the same SDS PAGE western blot. Panels C and D refer to the same blue native PAGE western blot. A) Blot was exposed for a short duration to visualize marker bands while avoiding over-exposure. B) Blot was exposed for a longer time to better visualize the annotated HCV envelope protein bands. C) As no blue native PAGE western blot marker was available the protein bands from a colorimetric native PAGE marker were marked with a pencil on the blot following transfer, but before ethanol washing. The pencil marks are clear in the colorimetric picture of the blot (panel C) and can be made out as faint white markings in the chemiluminescence capture (panel D). D) folded HCV E2 protein was probed on the blot using the conformational antibody, AR3A. (PDF) [file pone.0255336.s001.pdf]
